# Integrative analysis of genome-wide DNA methylation and single-nucleotide polymorphism identified ACSM5 as a suppressor of lumbar ligamentum flavum hypertrophy

**DOI:** 10.1186/s13075-021-02625-5

**Published:** 2021-09-30

**Authors:** Yanlin Cao, Yenan Zhan, Sujun Qiu, Zhong Chen, Kaiqin Gong, Songjia Ni, Yang Duan

**Affiliations:** 1 Department of Spine Surgery, Zhujiang Hospital, Southern Medical University, Guangzhou, 510280 China; 2 Department of Neurosurgery, Zhujiang Hospital, Southern Medical University, Guangzhou, 510280 China; 3 Department of Orthopaedic Trauma, Zhujiang Hospital, Southern Medical University, Guangzhou, 510280 China

**Keywords:** Genome-wide DNA methylation, Single-nucleotide polymorphism, Hypertrophy of ligamentum flavum, ACSM5, Cell fibrosis

## Abstract

**Background:**

Hypertrophy of ligamentum flavum (HLF) is a common lumbar degeneration disease (LDD) with typical symptoms of low back pain and limb numbness owing to an abnormal pressure on spinal nerves. Previous studies revealed HLF might be caused by fibrosis, inflammatory, and other bio-pathways. However, a global analysis of HLF is needed severely.

**Methods:**

A genome-wide DNA methylation and single-nucleotide polymorphism analysis were performed from five LDD patients with HLF and five LDD patients without HLF. Comprehensive integrated analysis was performed using bioinformatics analysis and the validated experiments including Sanger sequencing, methylation-specific PCR, qPCR and ROC analysis. Furthermore, the function of novel genes in ligamentum flavum cells (LFCs) was detected to explore the molecular mechanism in HLF through knock down experiment, overexpression experiment, CCK8 assay, apoptosis assay, and so on.

**Results:**

We identified 69 SNP genes and 735 661 differentially methylated sites that were enriched in extracellular matrix, inflammatory, and cell proliferation. A comprehensive analysis demonstrated key genes in regulating the development of HLF including ACSM5. Furthermore, the hypermethylation of ACSM5 that was mediated by DNMT1 led to downregulation of ACSM5 expression, promoted the proliferation and fibrosis, and inhibited the apoptosis of LFCs.

**Conclusion:**

This study revealed that DNMT1/ACSM5 signaling could enhance HLF properties in vitro as a potential therapeutic strategy for HLF.

**Supplementary Information:**

The online version contains supplementary material available at 10.1186/s13075-021-02625-5.

## Background

Lumbar degeneration diseases (LDDs) are common spinal lesions with typical symptoms of low back pain and limb numbness owing to an abnormal pressure on spinal nerves [1]. LDDs limit the patients’ mobility immensely and decrease their life quality significantly. It is worth noting that LDDs mostly occur in the elderly population, which brings high pressure on the whole society, especially with the rapid population aging in the major countries. LDDs are mainly composed of lumbar spinal canal stenosis (LSCS), degenerative lumbar spondylolisthesis (DLS), lumbar disc herniation (LDH), of which LSCS is most familiar with a prevalence of 47% in patients with legs pain and numbness. Among the causative factors, ligamentum flavum hypertrophy (HLF) is thought to be the most critical factor in the pathogenesis of LSCS [2, 3]. However, the ligamenta flava in LSCS patients are often 4-8 mm, compared with less than 4 mm in normal ligamenta flava [4].

Ligamenta flava consist of 80% elastic fibers and 20% collagen fibers [5], which makes them able to connect the adjacent vertebrae, keep vertebrae’ mobility, and protect the spinal cord [6]. As we know, lumbar suffers almost the whole weight of the upper body. Similar to that, lumbar ligamenta flava also bear the highest pressure among all the ligamenta flava in the vertebral column. Because of the high loads, lumbar ligamenta flava have a significantly higher frequency of becoming injured, especially the lowest L4/5 lumbar ligamentum flavum [7]. The injuries will further lead to fibrotic changes with a loss of elastic fibers and a cumulation of collagen fibers [8]. And it has been reported that fibrosis may be the primary pathology of HLF [9].

Previous studies have demonstrated some molecular mechanisms underlying HLF, for example, inflammatory pathways including TGF-β1, COX-2/PGE2, and iNOS; JNK pathway; p38 pathway; and angiogenesis processes including Angptl2 and VEGF [10–13]. However, the mechanisms behind HLF are still poorly understood and lack global screens. As we know, mechanical stress may be the primary causative factor for HLF, and mechanical stress has a great relationship with the habits and the environment. Furthermore, the critical influence environment on the epigenetics has been well established and thus affects the gene expression profoundly [14]. So, we hypothesize epigenetics may play an essential role in HLF.

Here, we investigated the epigenetic changes in the LDD patients with or without HLF (5 patients in each group) by the use of genome-wide DNA methylation and single-nucleotide polymorphism (SNP) analysis to widen our understanding of HLF in a global field. We identify the methylation levels of COL4A3, HSPA6, PIGC, and ACSM5 had positive correlation with HLF. Then, we found DNMT1-mediated hypermethylation of ACSM5 promoter leads to downregulation of ACSM5 expression in LFCs from HLF patients, and ACSM5 inhibits the proliferation and fibrosis and promotes the apoptosis of LFCs from HLF patients. Finally, we demonstrated that inhibition of DNMT1 suppressed HLF properties via regulation of ACSM5 expression.

## Materials and methods

### Human ligamentum flavum sample collection

Human ligamentum flavum samples in this study were collected according to protocols approved by the Ethical Committee of the Zhujiang Hospital of Southern Medical University (Guangzhou, China). Informed consents were obtained from all subjects. Ligamentum flavum samples were isolated from the anatomical region (L4/5) of LDD patients. Ligamentum flavum samples acquired from LDD patients with ligamentum flavum thickening (≤ 4 mm) were considered to be the non-ligamentum flavum hypertrophy (non-HLF) group, whereas the pathological ligamentum flavum samples harvested from LDD patients with ligamentum flavum thickening (> 4 mm) were assigned to the HLF group. The patients’ clinical characters are shown in Table 1.

**Table 1:** Clinical data of patients in two groups

Item	Sequencing of genome-wide SNP and DNA methylation			Verification of genome-wide SNP and DNA methylation		
	non-HLF (*n* = 5)	HLF (*n* = 5)	*P* value	non-HLF (*n* = 10)	HLF (*n* = 10)	*P* value
Age/years	58 ± 5	75 ± 5	5.80E-02	52 ± 5	69 ± 3	2.00E-02
LF thickness/cm	0.28 ± 0.01	0.52 ± 0.03	6.12E-05	0.31 ± 0.01	0.52 ± 0.02	6.29E-09
Gender	3F/2M	4F/1M		5F/5M	8F/2M	
Lumbar level	L4/5	L4/5		L4/5	L4/5	

Data are presented as the mean ± S.E.M.. *P* < 0.05 is considered to be significant. LF, ligamentum flavum. Non-HLF, LDD patients without ligamentum flavum hypertrophy (LF thickness ≤ 4 mm). HLF, LDD patients with ligamentum flavum hypertrophy (LF Thickness > 4 mm). F/M, female/male

### DNA extraction

Ligamentum flavum samples and cultured ligamentum flavum cells were used for DNA extraction with a QIAamp DNA Midi Kit (Qiagen). Extracted DNA was digested with RNase (Thermo Scientific) and purified with a Genomic DNA Clean Kit (Zymo Research).

### Whole-exome sequencing (WES)

WES was performed as previously described [15]. Briefly, Agilent SureSelect All Exon V6 (Agilent Technologies) with reagents were used for the synthesis of the library. Sequencing was performed on an Illumina HiSeq 2500 platform (Illumina).

### Single-nucleotide polymorphism (SNP) detection and analysis

The raw data of WES were performed QC process with fastqc and removed adapters with Cutadapter 2.10. Then, the clean data were mapped to the human reference genome GRCh37/hg19 by BWA-MEM. We used SAMtools for SNP detection and ANNOVAR for SNP annotation.

Detected SNPs were scored by SIFT, CADD, and Polyphen2_HVAR [16–18]. SNPs with scores > 0.05 in SIFT, > 15 in CADD, and < 0.909 in Polyphen2_HVAR were filtered.

### Infinium Methylation EPIC BeadChip assay

The methylation assays were performed the same as described previously [19]. Genomic DNA was sulfurated with the EZ DNA Methylation kit (Zymo Research). Genome-wide DNA methylation profiles were generated by Illumina’s Infinium MethylationEPIC BeadChip assay (EPIC array) (Illumina), determining > 850,000 methylated sites. The assay was scanned on an Illumina HiScan.

### EPIC array data processing

Raw EPIC array data were performed with CHAMP, an R package. Filtered data were used for evaluating the methylation level of each methylated site with a *β* value. A site with a *β* value > 0.6 was considered an up-methylated site, whereas a site with a *β* value < 0.2 was considered a down-methylated site. Then, the data were normalized using Illumina’s default normalization method. Normalized data were used for the detection of differentially methylated sites between HLF group and CT group with an R package “limma.” In detail, a linear model was established to calculate the *P* value of each site, and the *P* value was subsequently adjusted with a Benjamini & Hochberg method. A site with a *P* value < 0.05 was considered as a differentially methylated site.

The Volcano plot, heatmap, Manhattan plot, and pie chart to illustrate the differentially methylated sites were drawn with an R package “ggplot2”.

### GO and KEGG pathway enrichment analysis

Differential genes were mapped to the GO database or KEGG database, and the enrichment analysis of GO term or KEGG pathway was performed using the function Clusterprofiler in R package [20]. The results are shown in figures drawn with ggplot2 or Cytoscape 3.7.2.

### Protein-protein interaction (PPI) analysis

PPI analysis was performed using STRING database (https://string-db.org/). The network was established with a parameter of score > 900. Established gene network was plotted using Cytoscape 3.7.2.

### Validation of the SNPs

The SNPs to be validated were chosen based on the WES data. The primers used for PCR were listed in Table 2. The PCR products were sequenced using Sanger method by Invitrogen.

**Table 2:** Primer sequences used for SNP verification

Gene	Forward primer	Reverse primer
COL4A3 rs6436669	AGGCATGTGCCACTGTACTG	CTGAGAGTGAAGAAGAGGACGA
COL4A3 rs10205042	TGCACTTCACTGTACTGTGGTT	GGTTTGTTGCCCGTGGTTTG
MACC1 rs975263	CCCGAGGAGAGCTATTGTGTCC	AGCAGTTGGAAGCAGGTGAAGT
PIGC rs1063412	ACCACAATGACATGCTGCTTCC	ATGCTTGGCATCACGTCTTCC
PIGC rs2230471	ACTAGGGCACTCTTCAGGTCAG	AGGTCAAGTGGCAGAAGGTCTT
ACSM5 rs8062344	CTCTAGTGCCTGTGGTGTTTGC	CCCTCCTCTCACCTTCTTCCTT

### Methylation-specific analysis (MSP)

The methylation level of ACSM5, COL4A3, PIGC, and HSPA6 were validated using methylation-specific PCR [21, 22]. A methylation-independent assay with non-CpG including primers for the β-actin gene (ACTB) was used in order to verify DNA quality and to normalize results. Specificity and cross-reactivity of methylated and unmethylated primers (Table 3) were evaluated by using unconverted gDNA, SB-converted methylated and non-methylated DNA standards. Analytical sensitivity of qMSP assays was evaluated by using serial dilutions of SB-converted methylated and nonmethylated DNA standards and was found to be 0.1%. The assay efficiency (expressed as *E* = 10^− 1^/^slope^ − 1) was evaluated by using serial dilutions of the SB-converted methylated DNA standards in H2O and was in the range of 91–105%. The analysis was performed according to the RQ sample (Relative Quantification) = 2^−ΔΔCT^ method. Specifically, ΔΔCT values were generated for each target after normalization by ACTB values and using 1% methylation as calibrator and then were multiplied by 100 (RQ = 2^−ΔΔCT^× 100).

**Table 3:** Primer sequences used for MSP

Gene	Forward primer	Reverse primer
ACSM5 M primer	CGAGGTTAGGAGATCGAGATC	AATCGCCATCTAATCGAAAA
ACSM5 U primer	TTATGAGGTTAGGAGATTGAGATT	AATCACCATCTAATCAAAAAAAA
COL4A3 M primer	GAATAAGCGGGGTTTTTC	CTTCTACCCGAACATCGTAC
COL4A3 U primer	GGAATAAGTGGGGTTTTTT	CCTTCTACCCAAACATCATAC
HSPA6 M primer	AGAGGGGGTCGATTTTTC	TTCGAATTTCCGCTAAAAAC
HSPA6 U primer	GAGAGAGGGGGTTGATTTTTT	TTCAAATTTCCACTAAAAACCAA
PIGC M primer	TGTAGTTATTTAGGAGTTTAAGGCGA	AAAAAAAATAATAACGAAACCCGTT
PIGC U primer	GTAGTTATTTAGGAGTTTAAGGTGA	AAAAAAAATAATAACAAAACCCATT
MACC1 M primer	TTGTATAATAGAATAGTAAAAATAAAATGC	TCTATCAATCTTTCAAAAAACGAT
MACC1 U primer	TTGTATAATAGAATAGTAAAAATAAAATGT	TTCTATCAATCTTTCAAAAAACAAT
ACTB primer	TGGTGATGGAGGAGGTTTAGTAAGT	AACCAATAAAACCTACTCCTCCCTTAA

*M primer* methylation primer, *U primer* non-methylation primer

### Correlation analysis and ROC analysis

The correlations of the key genes hypermethylation and flavonoid ligament hypertrophy was analyzed by Pearson correlation. The coefficient of association and *P* value were performed to confirm their association. The ROC curves were analyzed by pROC package in R based on the gene methylation level and the patients’ clinical characters.

### Isolation and culture of ligamentum flavum cells

To extract of human ligamentum flavum cell, ligamentum flavum samples were isolated from LDD patients with or without ligamentum flavum hypertrophy. Then, the samples were minced and digested with 0.2% type I collagenase 2 for 1 h at 37 °C. Digested tissues were seeded in 6-cm dishes and cultured in DMEM medium (Lonza) containing 10% fetal bovine serum (Gibco) and 1% penicillin/streptomycin (Invitrogen) for 7 days. Migrated cells were digested and pass on for subsequent experiments.

The expression levels of collagen I (Abcam, ab260043), collagen III (Abcam, ab184993), vimentin (Abcam, ab16700), and fibronectin (Abcam, ab2413) were detected by immunofluorescence staining and observed under a Leica Microsystems DMI4000 fluorescence microscope with a DFC365FX camera (Leica Microsystems) to identify the cultured ligamentum flavum cells (LFCs).

### Transfection and cell treatment

The pCMV-DNMT1, pCMV-ACSM5, and pCMV were purchased from Hanbio Biotechnology. sh-DNMT1, sh-ACSM5, and sh-NC were purchased from Thermofisher Biotechnology. Transfection of plasmid and shRNAs was performed using Lipo2000 (Thermofisher) according to the protocol of the manufacture. The sequences for sh-DNMTs and sh-ACSM5 are shown in Table 4.

For the treatment of 5-AzaC, 0 μM, 1 μM, 5 μM, and 10 μM 5-AzaC were added to LFCs from HLF tissues for 24 h, respectively. Then, the cells were used for other experiments.

**Table 4:** The primer sequences of RT-PCR and the sequences of shRNAs

Gene	Sequences (5′-3′)
ACSM5	Forward primer: ACACTGGCTGGGTGAAGG Reverse primer : ACAGCAGAGGGTGGTTATCG
COL4A3	Forward primer: AGCAAGGGTTGTGTCTGTAAAG Reverse primer :CAGAAAATCCTGGCAATCCACT
HSPA6	Forward primer: CAGTCAGATCATCTTCTCGA Reverse primer :CTTCCATGAAGTGGTTCACGA
MACC1	Forward primer: CATTTTCGGTCAGGAAGAATTGC Reverse primer :TGGAAGCATTATTACCACGAAGG
PIGC	Forward primer: GCCCCGAAGCGGGAAAAAG Reverse primer :CAACACAGCCAAGTTCCCGA
GAPDH	Forward primer: AAGTATGACAACAGCCTCAAG Reverse primer :TCCACGATACCAAAGTTGTC
DNMT1 shRNA	CCGGGACGACCCTGACCTCAAATATCTCGAGATATTTGAGGTCAGGGTCGTCTTTTTG
DNMT3A shRNA	CCGGCCCAAGGTCAAGGAGATTATTCTCGAGAATAATCTCCTTGACCTTGGGTTTTTG
DNMT3B shRNA	CCGGAGTGCCGACAGCTCTCCAATACTCGAGTATTGGAGAGCTGTCGGCACTTTTTTTG
ACSM5 shRNA	CCGGGATGTGCAGATTGTGGATGATCTCGAGATCATCCACAATCTGCACATCTTTTTTG

### CCK8 assays

Cells (5000 cells/well in 96-well plates) were adjusted to different treatments for 24–72 h at 37 °C. Then, 10 μl CCK-8 reagent (Abcam) was added to each well and cultured for another 1 h at 37 °C. The absorption values were measured by a microplate reader (Thermofisher) and were used to calculate the cell viability.

### Apoptosis assays and the test of caspase 3 activity

Cells (5 × 10^5^ cells/well in 6-well plates) were adjusted to different treatments for 24 h at 37 °C. Then, the cells were digested and stained with an Annexin V-FITC/propidine iodide (PI) double-staining kit (Abcam) according to the manufacturer’s instructions. The cells were then analyzed with FACS using CytoFlex (Beckman).

Caspase 3 activity was measured with a caspase 3 assay kit (Abcam) according to the manufacturer’s instructions.

### RT-PCR analysis

Total RNA was extracted with TRIzol (Invitrogen) according to the manufacturer’s protocol. The synthesis of cDNA was used for the mRNA Reverse Transcription Kit (Qiagen). Amplification reactions was performed with SYBR Green PCR Master Mix (Applied Biosystems) using a CFX96 Real-Time System thermocycler (Bio-Rad) and normalized to GAPDH. The relative expression of genes were calculated using the 2^−△△Ct^ method. All the primers’ sequences were listed in Table 4.

### Western Blot analysis

The procedure of western blot was the same as the previous paper [23]. In brief, the total protein from the cells was lysed with radio immunoprecipitation assay buffer (Solarbio Biotech). Protein concentrations were measured using BCA protein assay kit (Beyotime) according to the manufacturer’s instructions. Then, equivalent amount of protein were separated by SDS-PAGE and transferred into polyvinylidene fluoride membranes (Bio-Rad). After blocking with 5% non-fat milk for 1 h at room temperature, the membranes were incubated with primary antibodies at 4 °C overnight and then incubated with secondary antibodies for 2 h at room temperature. Bands were visualized with an ECL detection kit (Thermofisher) using ChemiDoc XRS Plus (BioRad) and analyzed using the Image J software. All primary antibodies and secondary antibodies in the study are listed as follows: anti-ACSM5 antibody (1:500, Proteintech), anti-collagen I (1:1000, Abcam), anti-collagen III (1:1000, Abcam), anti-MMP2 (1:1000, Proteintech), anti-MMP13 (1:1000, Proteintech), and anti-β-actin antibody (1:5000, Abcam).

### Statistical analysis

Data are shown as the mean ± S.E.M. Statistical analyses of data were performed using GraphPad 6.0 Software. Analyses of significant differences between groups were performed using Student’s *t* tests or ANOVA with Tukey’s test.

## Results

### SNP and DNA methylation profiles in HLF

We collected five L4/5 ligamentum flavum samples from LDD patients with HLF (HLF group) and five samples in the same location from LDD patients without HLF (CT group). The detailed clinical characters of the patients are shown in Table 1. Each sample was separated into two and performed whole-exome sequencing (WES) and Illumina Infinium MethylationEPIC BeadChips assays (EPIC arrays).

**Fig. 1:**
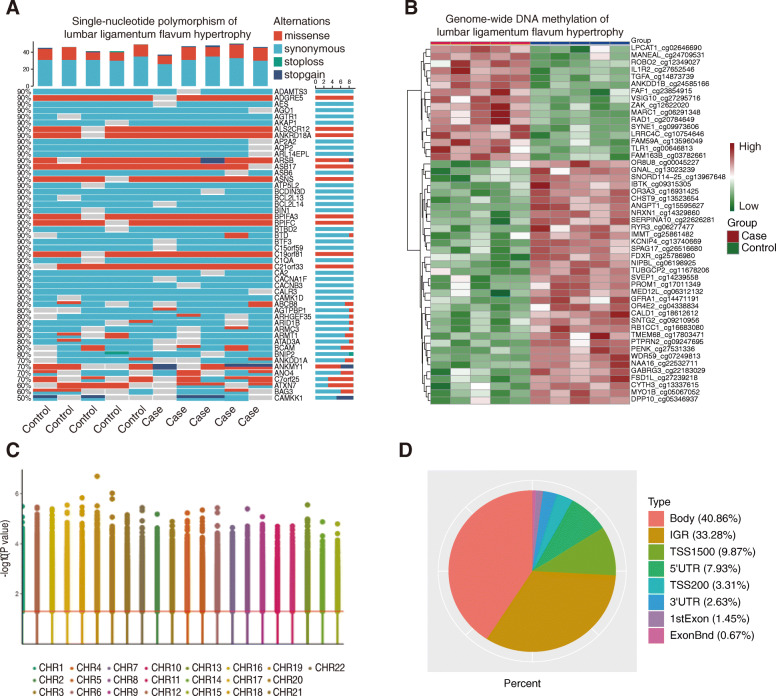
Genome-wide SNP and DNA methylation analysis between the HLF group and the CT group. **A** Waterfall graph of the SNPs. The *x*-axis represents the sample names, of which the first five belong to the CT group and the ladder five belong to the HLF group. The left *y*-axis represents the percentage of the major SNP type in the total ones. The right *y*-axis represents the gene names. The colors represent the SNP types. Red means missense; light blue means synonymous; green means stoploss; and dark blue means stopgain. **B** Heatmap showing the top 50 differentially methylated sites. **C** Manhattan plot showing the distribution of the differentially methylated sites. **D** Pie chart showing the composition of the differentially methylated sites’ locations among the genome. “Body” represents genes’ introns and exons. “IGR” represents the intergenic region. “TSS 1500” represents 1500 bp ahead of the transcription start site (TSS). “TSS 200” represents 200 bp ahead of the transcription start site (TSS). “UTR” represents the untranslated region. “ExonBnd” represents the border of the exons

We used SAMtools to identify the SNPs and ANNOVAR to annotate the SNPs from the WES data. Then, we evaluated the SNPs using SIFT (> 0.05), CADD (> 15), and Plyphen2_HVAR (< 0.909), and we got 69 SNP genes with significance compared to the reference genome sequence (Fig. 1A). We found broad missense SNPs among all the 69 genes, indicating they might probably participate in the development of HLF. After quality control and filtering, a total of 735,661 differentially methylated sites were identified, of which 415,237 were up-methylated sites and 320,424 were down-methylated (Supplementary Fig. 1). Heatmap of the top 50 differentially methylated sites shown a significant preference with up-methylated sites (45 up-methylated sites, contrary to 5 down-methylated sites; Fig. 1B). The differentially methylated sites were equally distributed among the autosomes (Fig. 1C). However, most of the differentially methylated sites were located at the gene body, with a prevalence of 40.88%. Furthermore, we have noticed that about 20% of the differentially methylated sites were at the promoter region, of which 9.87% were at the area 1500 bp up to the transcription start site (TSS) and 7.93% were at the 5' UTR (Fig. 1D). Methylation changes at either the gene body or promoter may influence the gene expression immensely. Therefore, the majority of the differentially methylated sites between the HLF group and the CT group might play an important role in regulating the signal pathways involved in the HLF.

To verify which bio-functions or pathways might be regulated by the differentially methylated sites, we performed GO (Gene Ontology) and KEGG (Kyoto Encyclopedia of Genes and Genomes) enrichment analysis. The GO terms consist of three themes: biology process (BP), cell component (CC), and molecular function (MF). The top 15 enriched terms in each analysis are shown in Supplementary Fig. 2. In summary, the bio-functions or pathways that were influenced by the differentially methylated sites were enriched in ECM-related terms, inflammation-related terms, and cell proliferation-related pathways including wnt pathway, Akt pathway, Ras pathway, and MAPK pathway. These findings are consistent with previous studies, like ECM, inflammation, and cell proliferation, which are thought to be the main causative factors for HLF [10–13].

### Integrated analysis of SNP and methylation data

**Fig. 2:**
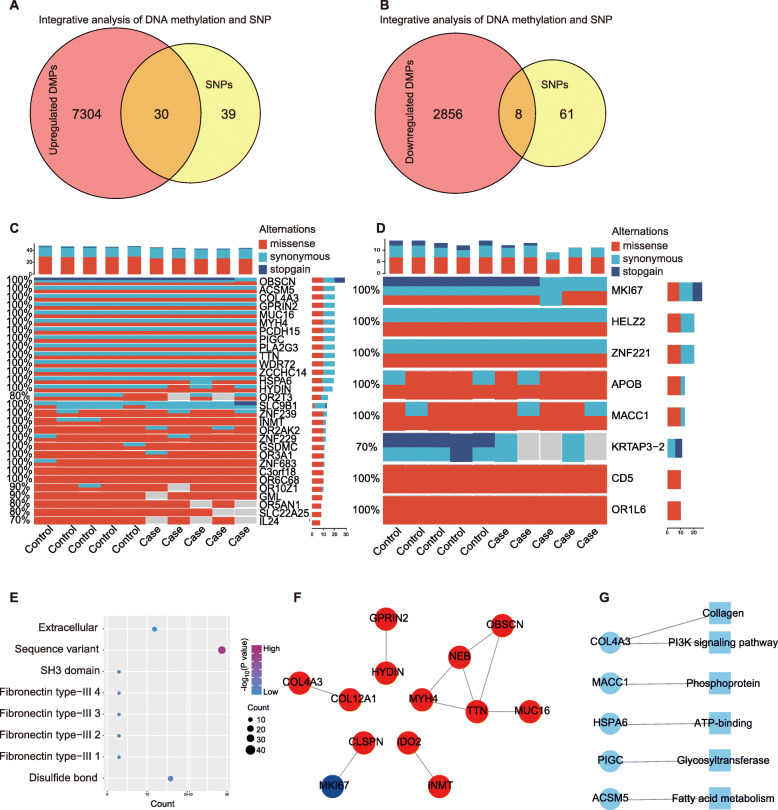
Combination analysis of the SNP genes and differentially methylated genes. **A** Statistical result revealing the intersection of the up-methylated genes and the SNP genes. **B** Statistical result revealing the intersection of the down-methylated genes and the SNP genes. **C** Waterfall graph showing the intersection of the up-methylated genes and the SNP genes. **D** Waterfall graph showing the intersection of the down-methylated genes and the SNP genes. **E** Bubble chart showing functional categories of the 38 crossed genes between the differentially methylated genes and the SNP genes. **F** A network showing the PPI analysis result of the 38 crossed genes. **G** A network showing the KEGG enrichment result of the 13 PPI genes

To investigate the HLF-causative genes, we combined the SNP and methylation data for integrated analysis. The 7334 up-methylated genes or the 2864 down-methylated genes were taken intersection with the 69 SNP genes, respectively. Therefore, we got 30 up-methylated SNP genes (Fig. 2A, C) and eight down-methylated SNP genes (Fig. 2B, D). Further functional categories enrichment analysis revealed that the 38 intersection genes were mainly enriched to sequence variant and Fibronectin type-III related pathways (Fig. 2E).

To ulteriorly verify the key genes that participate in the process of HLF in the 38 filtered genes, we performed protein-protein interaction (PPI) analysis using STRING database with a filtered threshold of score > 900. The result indicated a network of following genes: CLSPN, COL12A1, GPRIN2, IDO2, MUC16, MYH4, NEB, OBSCN, MKI67, COL4A3, HYDIN, INMT, and TTN (Fig. 2F). Meanwhile, we mapped the 38 candidates to the KEGG database and filtered out five genes: COL4A3, MACC1, HSPA6, PIGC, and ACSM5. These five genes were all up-methylated genes and mainly participated in glucose and lipid metabolism, PI3K pathway, collagen related pathways, and so on (Fig. 2C, D, G).

Combined those results above, we propose that COL4A3, MACC1, HSPA6, PIGC, and ACSM5 might be the key genes in the process of HLF. And the relationship of those genes with HLF has never been reported.

### Methylation and SNP verification of the key genes in clinical samples

We collected ten L4/5 ligamentum flavum samples from LDD patients with HLF (HLF group) and ten samples in the same location from LDD patients without HLF (non-HLF group). The clinical characters of the patients were shown in Table 1.

**Fig. 3:**
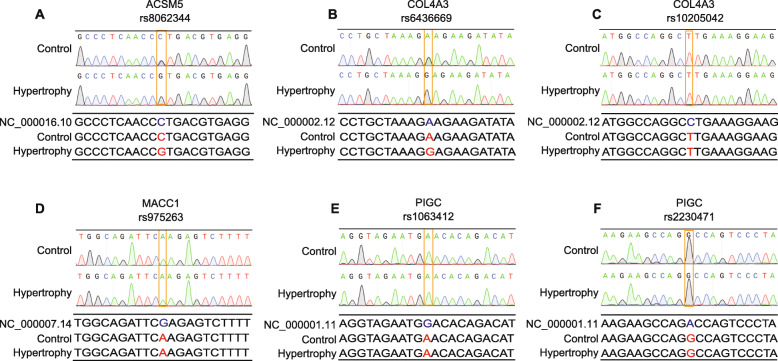
Validation of the SNP sites in indicated genes. **A** Sanger sequencing result of rs8062344 (at ACSM5) in the HLF and CT group compared with the reference genome. **B** Sanger sequencing result of rs6436669 (at COL4A3) in the HLF and CT group compared with the reference genome. **C** Sanger sequencing result of rs6436669 (at COL4A3) in the HLF and CT group compared with the reference genome. **D** Sanger sequencing result of rs975263 (at MACC1) in the HLF and CT group compared with the reference genome. **E** Sanger sequencing result of rs1063412 (at PIGC) in the HLF and CT group compared with the reference genome. **F** Sanger sequencing result of rs2230471 (at PIGC) in the HLF and CT group compared with the reference genome

To verify the SNP sites of the five genes, we extracted DNA from the patients' samples and performed DNA sequencing with the Sanger method. For COL4A3 (rs10205042), MACC1 (rs975263), and PIGC (rs2230471 and rs8062344), the verification data were the same with the WES data (Fig. 3C–F). For ACSM5 (rs 8062344) and COL4A3 (rs6436669), only the HLF samples had the same SNPs with our WES data, while the non-HLF samples were the same with the reference genome (Fig. 3A, B). Those data revealed that the majority of verified results were consistent with our WES data.

**Fig. 4:**
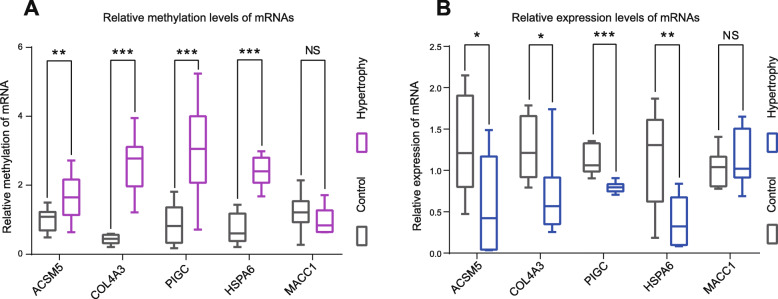
Validation of the methylation levels of ACSM5, COL4A3, PIGC, and HSPA6 using MSP. Data were presented by mean ± SEM (*n* = 10). ^*N.S.*^*P* > 0.05, **P < 0.01, ***P < 0.001

To validate the methylation level, we performed methylation-specific PCR (MSP). ACSM5, COL4A3, PIGC, and HSPA6 showed immensely increased methylation levels in the HLF group compared to the non-HLF group, consistent with our genome-wide DNA methylation data (Figs. 1B, 2c, 4). However, MSP results showed no methylation difference of MACC1 between the HLF group and the non-HLF group (Fig. 4).

**Fig. 5:**
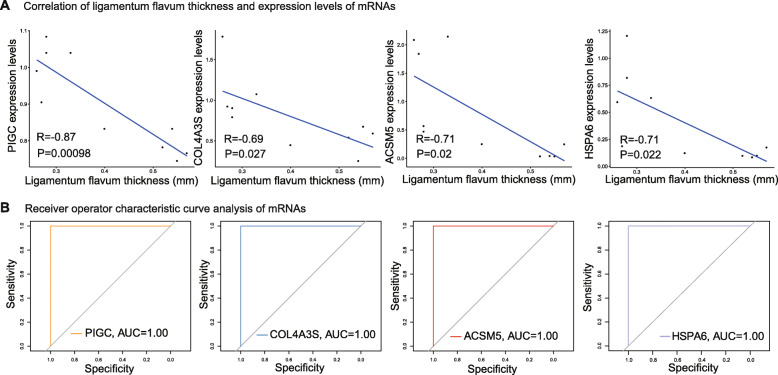
Analysis of the relationship between indicated genes’ methylation level and clinical HLF characters. **A** Correlation analysis of ligamentum flavum thickness with COL4A3, HSPA6, PIGC, and ACSM5’s DNA methylation level, respectively. The *x*-axis represents the gene’s methylation level; the *y*-axis represents ligamentum flavum thickness. **B** ROC analysis of HLF with COL4A3, HSPA6, PIGC, and ACSM5’s DNA methylation level, respectively

Then, we explored the relationship between the genes’ methylation level with the patients’ clinical characters. We investigated the correlation between the methylation levels of COL4A3, HSPA6, PIGC, or ACSM5 with the patients’ ligamentum flavum thickness and performed ROC analysis to evaluate the clinical efficiency and sensitivity of the indicated genes. We found that the methylation levels of COL4A3, HSPA6, PIGC, and ACSM5 were all positively correlated with the patients’ ligamentum flavum thickness (correlation index arrange from − 0.69 to − 0.87; Fig. 5A). And it is expected that the four genes all shown perfect efficiency and sensitivity for HLF with AUC values of 1.00 (Fig. 5B).

Therefore, we validated the SNP status and methylation levels of COL4A3, MACC1, HSPA6, PIGC, and ACSM5. The results were mostly consistent with the data of our genome-wide DNA methylation and SNP analysis. Furthermore, we found that the methylation level of COL4A3, HSPA6, PIGC, and ACSM5 had a perfect sensitivity for HLF, highlighting their therapeutic potential for HLF.

### DNA methylation downregulated the expression of ACSM5 in a DNMT1-dependent manner

**Fig. 6:**
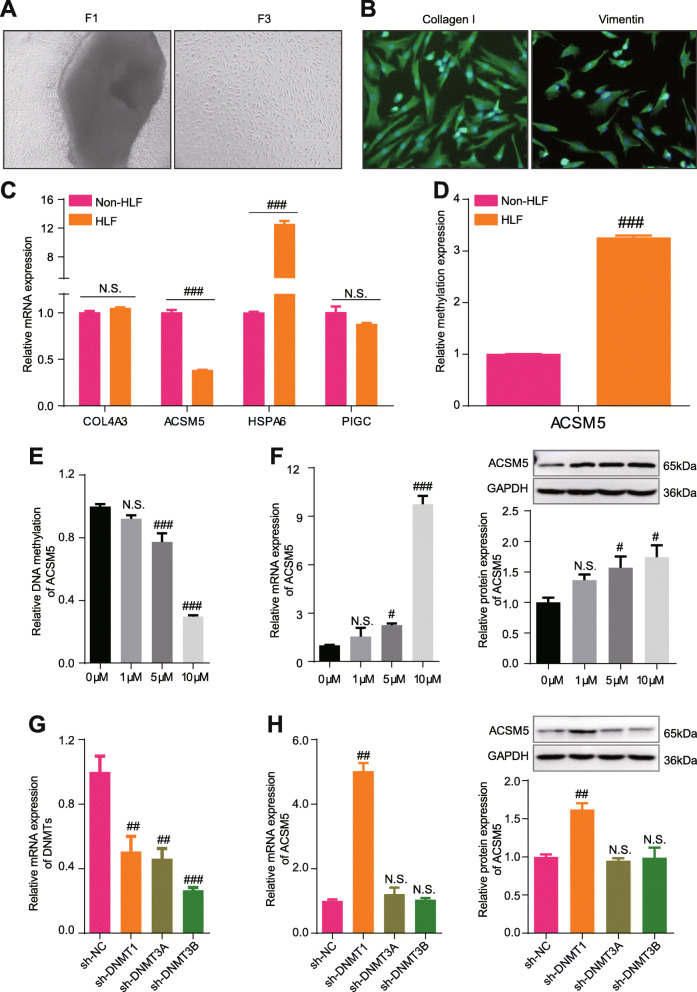
DNA methylation downregulated the expression of ACSM5 in a DNMT1-dependent manner. **A** Pictures showing the morphology of isolated LFCs with 1st and 3rd passage. **B** Identification of the phenotype of cultured ligamentum flavum (LF) cells using immunofluorescence staining. Immunofluorescence staining for collagen I and Vimentin in cultured cells; *n* = 3 experiments. **C** The relative expression level of COL4A3, HSPA6, PIGC, and ACSM5 compared between LFCs from the HLF group and LFCs from the non-HLF group. **D** The relative DNA methylation level of ACSM5 compared between LFCs from HLF group and LFCs from non-HLF group. **E** The relative DNA methylation level of ACSM5 in LFCs from HLF patients with the treatment of different concentrations of 5-AzaC. **F** qPCR and Western Blot results showing the expression level of ACSM5 in LFCs from HLF patients with the treatment of different concentrations of 5-AzaC. **G** The relative expression level of DNMTs in LFCs from HLF patients with the treatment of indicated DNMT’s shRNA. **H** qPCR and Western Blot results showing the expression level of ACSM5 in LFCs from HLF patients with the treatment of indicated DNMT’s shRNA. Data were presented as the mean ± SME of three independent experiments. ^*N.S.*^*P* > 0.05, ^#^*P* < 0.05, ^##^*P* < 0.01, ^###^*P* < 0.001

To explore the therapeutic potential of COL4A3, HSPA6, PIGC, and ACSM5, we performed functional experiments in vitro. We isolated ligamentum flavum cells (LFCs) from ligamentum flavum samples belonged to LDD patients with HLF (HLF group) or without HLF (non-HLF group). LFCs of the 3rd passage were identified through immunofluorescence staining (Fig. 6A, B and Fig.S4) and used for subsequent experiments. The cultured cells had a typical LFCs phenotype and uniformly expressed of LFCs markers (collagen I, collagen III, vimentin or fibronectin) in each cell, suggesting the high purity of the LFCs obtained. The expression of COL4A3, HSPA6, PIGC, and ACSM5 in HLF cells and non-HLF cells was first examined by qPCR. The results showed that HLF, ACSM5 was downregulated, HSPA6 was upregulated, and COL4A3 and PIGC had no change in HLF cells (Fig. 6C). Commonly, a higher level of DNA methylation brings a decrease in the gene expression; however, other factors may diminish or even converse this phenomenon [24, 25], indicating the regulation of COL4A3, HSPA6, and PIGC’s expression might be complex while ACSM5’s expression was mainly influenced by DNA methylation. Then, we validated that ACSM5 had a higher DNA methylation level in HLF group (Fig. 6D). Furthermore, decreasing DNA methylation levels were observed with the treatment of increasing doses of 5-AzaC (Fig. 6E) and liberated the expression of ACSM5 in the HLF group (Fig. 6F), proving the solid relationship between DNA methylation level and the expression of ACSM5.

As we know, DNA methylation is mainly regulated by the DNMT family. To explore the upstream regulators of ACSM5’s DNA methylation, we knocked down DNMT1, DNMT3A, and DNMT3B in HLF cells (Fig. 6G) and found that only knocking down of DNMT1 could rescue the expression level of ACSM5 (Fig. 6H), indicating the methylation of ACSM5 was mainly contributed by DNMT1. Nevertheless, overexpression of DNMT1 could decrease the expression of ACSM5 (Supplementary Fig. 3). The above results indicate that the downregulation of ACSM5 in HLF is related to its promoter hypermethylation, which is regulated by DNMT1.

### ACSM5 regulates the proliferation, fibrosis, and apoptosis of LFCs

**Fig. 7:**
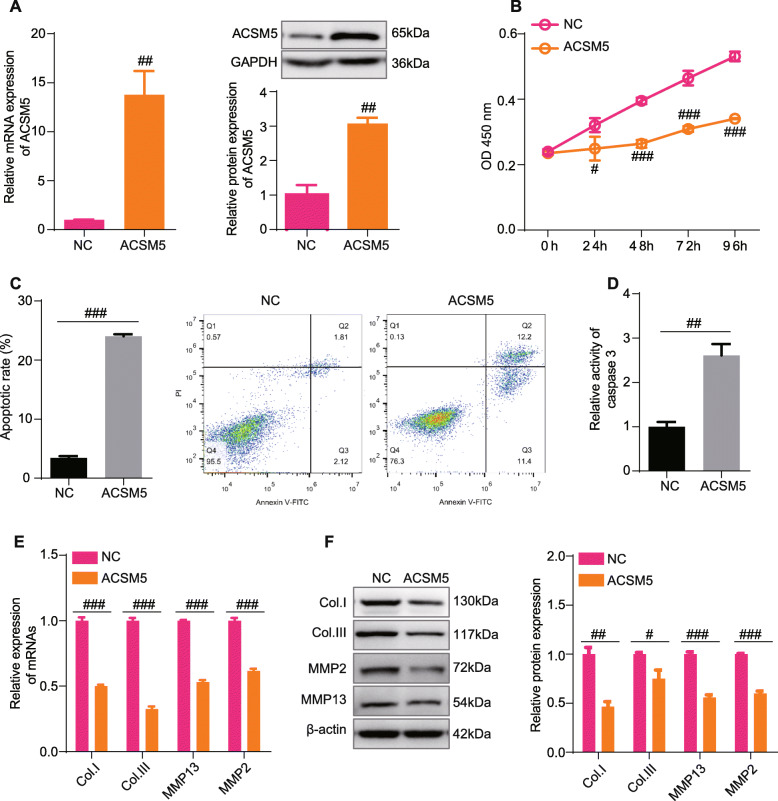
ACSM5 regulates the proliferation, fibrosis, and apoptosis of LFCs. **A** qPCR and Western Blot results showing the expression level of ACSM5 in LFCs from HLF patients with the treatment of pCMV-ACSM5 or empty plasmid. **B** CCK8 assays of LFCs from HLF patients with the treatment of pCMV-ACSM5 or empty plasmid. Absorbances were measured at 24 h, 48 h, 72 h, and 96 h post cell seeding. **C** Flow graph and statistical results of apoptosis assays using LFCs from HLF patients with the treatment of pCMV-ACSM5 or empty plasmid. **D** The relative caspase3 activity of LFCs from HLF patients with the treatment of pCMV-ACSM5 or empty plasmid. **E** qPCR results showing the expression level of COL1, COL3, MMP13, and MMP2 in LFCs from HLF patients with the treatment of pCMV-ACSM5 or empty plasmid. **F** Western Blot results showing the expression level of COL1, COL3, MMP13, and MMP2 in LFCs from HLF patients with the treatment of pCMV-ACSM5 or empty plasmid. Data were presented as the mean ± SME of three independent experiments. ^#^*P* < 0.05, ^##^*P* < 0.01, ^###^*P* < 0.001

We over-expressed ACSM5 in LFCs from HLF patients (Fig. 7A) and found that the over-expression of ACSM5 could inhibit the proliferation of LFCs from HLF patients (Fig. 7B). At the same time, ACSM5 could also promote the apoptosis and enhance the activity of caspase3 in LFCs from HLF patients (Fig. 7C, D). Moreover, over-expression of ACSM5 could decrease the fibro features of LFCs from HLF patients, manifesting as decreasing expression of COL1, COL3, MMP13, and MMP2 (Fig. 7E, F). Collectively, these results demonstrated that ACSM5 could inhibit the proliferation and fibrosis and promote the apoptosis of LFCs from HLF patients.

### DNMT1 regulates the proliferation, fibrosis, and apoptosis of LFCs via downregulating ACSM5 expression

**Fig. 8:**
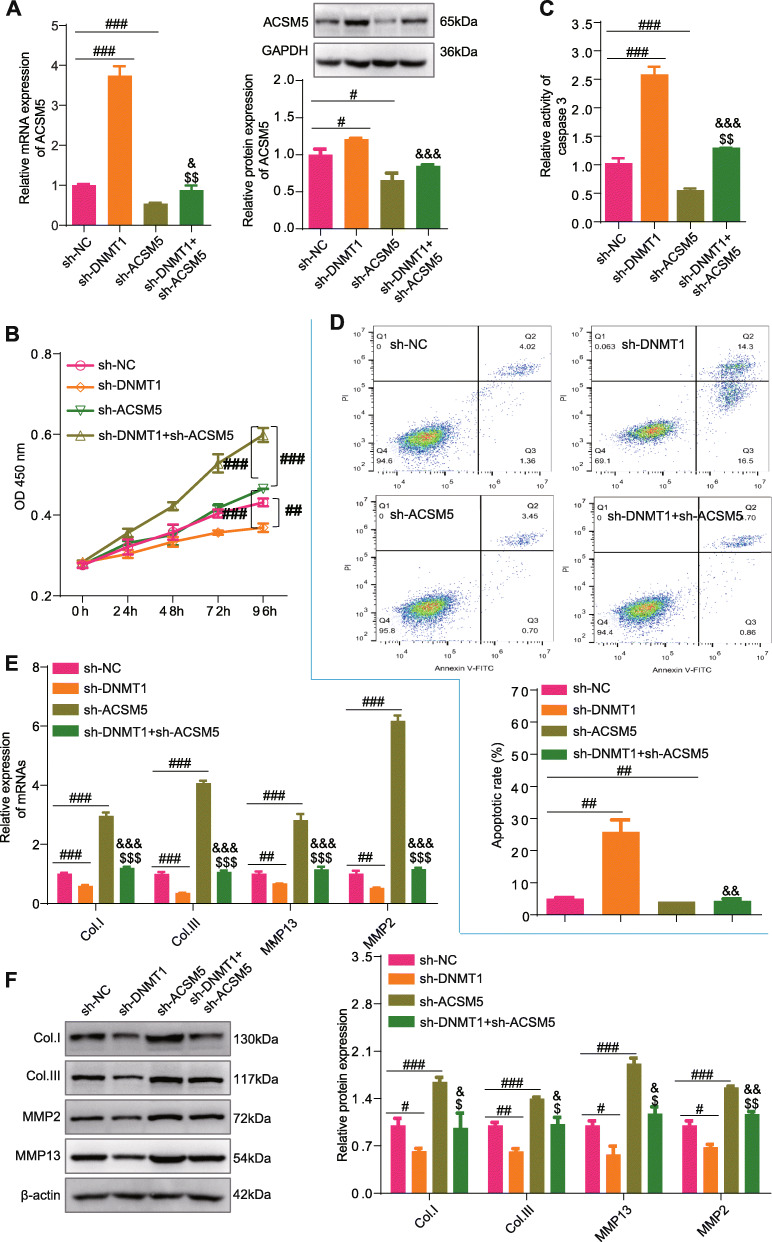
DNMT1 regulates the proliferation, fibrosis, and apoptosis of LFCs via downregulating ACSM5 expression. **A** The relative mRNA and protein expression level of ACSM5 in LFCs from HLF patients with the treatment of indicated shRNAs. **B** CK8 assays of LFCs from HLF patients with the treatment of indicated shRNAs. Absorbances were measured at 24 h, 48 h, 72 h, and 96 h post cell seeding. **C** The relative caspase3 activity of LFCs from HLF patients with the treatment of indicated shRNAs. **D** Flow graph and statistical results of apoptosis assays using LFCs from HLF patients with the treatment of indicated shRNAs. **E** RT-PCR results showing the expression level of COL1, COL3, MMP13, and MMP2 in LFCs from HLF patients with the treatment of indicated shRNAs. **F** Western Blot results showing the expression level of COL1, COL3, MMP13, and MMP2 in LFCs from HLF patients with the treatment of indicated shRNAs. Data were presented as the mean ± SME of three independent experiments. Tukey’s test, ^#^*P* < 0.05, ^##^*P* < 0.01, ^###^*P* < 0.001. Dollar sign ($) symbol represented the significance compared to the sh-ACSM5 group, ^$^*P* < 0.05, ^$$^*P* < 0.01, ^$$$^*P* < 0.001. Ampersand (&) symbol represented the significance compared to the shDNMT1 group, ^&^*P* < 0.05, ^&&^*P* < 0.01, ^&&&^*P* < 0.001

We have proved that knocking down of DNMT1 could increase the expression of ACSM5; however, knocking down of ACSM5 at the same time could diminish this influence (Fig. 8A). Then, we observed that knocking down DNMT1 could inhibit the proliferation, promote apoptosis, and decrease the fibro features of LFCs from HLF patients (Fig. 8B–F), which were similar to the influences of ACSM5 over-expression. Furthermore, knocking down of ACSM5 could promote the proliferation, inhibit apoptosis, and enhance the fibro features of LFCs from HLF patients; however, knocking down of DNMT1 at the same time could rescue the influence of knocking down of ACSM5 (Fig. 8B–F). Taken together, these data proved that the silencing DNMT1 suppresses the proliferation and fibrosis and promote the apoptosis of LFCs from HLF patients via regulating ACSM5 expression.

## Discussion

With the acceleration of global aging and changes in lifestyle, the incidence of LSCS increases year by year and tends to be younger, which has seriously threatened the health and life of patients [26]. Currently, HLF is thought to be a critical pathological leading to LSCS [27], but the precise molecular mechanisms of HLF remain unknown so far. Herein, we used the genome-wide DNA methylation sequencing and SNP sequencing for the first time to analyze the epigenetic changes in HLF tissues and non-HLF tissues. Integration analysis of the above two sequencing results would help us to identify the key molecules that regulate the occurrence and development of HLF. Fortunately, we found that five key genes may be involved in the occurrence and development of HLF, among which ACSM5 was hypermethylated and low expressed in HLF tissues and cells.

Previous studies have raised the importance of SNP to LDDs [28], but a genome-wide screen is lacking in HLF tissues in patients with LSCS. Our research revealed that the HLF tissues shared most SNPs with the non-HLF tissues, but there are also some different SNPs, which might be involved in the occurrence and development of HLF. In addition, DNA methylation is an important epigenetic mechanism. Studies have reported abnormal DNA methylation in ossified spinal LF tissues and calcified thoracic LF tissues [29, 30], but the differential expression profile of DNA methylation between HLF tissues and non-HLF tissues has not been reported. In this study, the whole genome epigenetic landscape of HLF and non-HLF was studied for the first time. We found that there were a large number of differential DNA methylation genes between HLF tissues and non-HLF tissues, and these differential DNA methylation genes were mainly enriched in MAPK, WNT, and PI3K/AKT signaling pathways, suggesting that these signaling pathways played an important role in the occurrence and development of HLF. Our findings were consistent with those of previous studies [27]. For example, Cao et al. reported that p38 MAPK signaling pathway promoted the homeostasis of the extracellular matrix and the hypertrophy of LF [31]. Zheng et al. indicated that the inhibitor of MAPK/ERK signaling pathway restrained TGF-1β-induced LF fibrosis, suggesting a crucial role of MAPK/ERK signaling pathway in HLF [32]. The involvement of WNT signaling pathway in HLF has been reported by Taiki et al. [33]. Moreover, Chen et al. demonstrated that estradiol upregulated the expression of MMP-13 via activating PI3K pathway and contributed to the homeostasis of ECM in the LF [34]. Zhou et al. revealed that LPA bind to the receptor LPAR1 to induce LF cells proliferation and anti-apoptosis through activating AKT signaling pathway [35].

We further integration analysis of DNA methylation sequencing data and SNP sequencing data to identify five key genes: COL4A3, HSPA6, PIGC, ACSM5, and MACC1. Validation of the SNP sites indicated that only ACSM5 rs80623344 and COL4A3 rs6436669 mutated in HLF tissues compared with non-HLF tissues. COL4A3 gene variants have been reported to be involved in the occurrence and development of various diseases, such as diabetic nephropathy [36], focal segmental glomerulosclerosis [37], and Alport syndrome [38, 39]. Here, it is the first time that COL4A3 gene mutation has been identified in HLF. In addition, ACSM5, as a member of acyl-CoA synthetase family, is located in mitochondrial matrix and involved in the first step of fatty acid metabolism, but physiological characteristics of ACSM5 have been seriously neglected by human beings [40]. Recently, the role of ACSM5 in human diseases has gradually attracted attention, and preliminary exploratory studies have been conducted in some fields. For example, studies have shown that ACSM5 is low expressed in tumor tissues of breast cancer and lung cancer and its expression level is related to the poor prognosis of patients, which can be used as a potential marker for disease prognosis and diagnosis [41, 42]. Qi et al. have found that ACSM5 is closely related to attention-deficit/hyperactivity disorder (ADHD), suggesting that ACSM5 may be a key target in the prevention and treatment of ADHD [43]. Cao et al. reported that ACSM5 was low expressed in renal tissue of rats with chronic glomerulonephritis (CGN), while Qiteng Xiaozhao granules significantly increased the low expression of ACSM5 in the renal tissue of rats with CGN, suggesting that ACSM5 was involved in the occurrence and development of CGN [44]. Cao et al. found that ACSM5 gene was hypermethylated in the peripheral blood of patients with myocardial infarction, and its methylation site and level might be closely related to the occurrence of myocardial infarction [45]. However, the role of ACSM5 in the HLF has not been reported yet. In this study, we identified for the first time that the low expression and hypermethylation of ACSM5 gene in tissues and cells of HLF, and overexpression of ACSM5 restrained proliferation, anti-apoptosis, and fibrosis of HLF cells in vitro. DNMT1, a type of DNA methyltransferases (DNMTs), can maintain and catalyze abnormal DNA hypermethylation of CpG islands to cause the loss expression of genes specific, thereby regulating the occurrence and development of various fibrosis-related diseases [46–48]. Our data demonstrated that the low expression of ACSM5 in HLF cells was negatively regulated by DNMT1, and suppression of DNMT1 restrained the proliferation, anti-apoptosis, and fibrosis of HLF cells in vitro, which can be reversed by silencing ACSM5. Taken together, our findings not only shed a new light on the mechanism of HLF, but also revealed that interference of DNMT1/ACSM5 signaling pathway might be a potential preventive and therapeutic strategy against patients with HLF.

However, there are certain limitations in this study. Firstly, the role of DNMT1/ACSM5 signaling pathway in the development of HLF has not been further verified in animal model with HLF. Secondly, the downstream signaling of ACSM5 in HLF remained largely unknown, which would be the focus of our future research.

## Conclusion

The present study revealed that positive correlations between the methylation level of COL4A3, HSPA6, PIGC, and ACSM5 and the development of HLF by the use of genome-wide DNA methylation and SNP analysis. Most importantly, we proved that downregulation of ACSM5 by DNMT1 could inhibit the proliferation and fibrosis, and promote the apoptosis of LFCs from HLF patients, shedding light on the therapeutic potential of DNMT1/ACSM5 signaling pathway.

## Supplementary Information

**Additional file 1: Supplementary Fig. 1** Volcano map showing the up-methylated sites and the down-methylated sites. The red dots indicate the methylation upregulation and the blue dots indicate the methylation downregulation in ligamentum flavum hypertrophy.

**Additional file 2: Supplementary Fig. 2** GO and KEGG enrichment analysis of the differentially methylated sites. Top 15 bio-functions or pathways enriched of the differentially methylated sites in GO_BP, GO_CC, GO_MF, and KEGG were shown, respectively.

**Additional file 3: Supplementary Fig. 3** qPCR and Western Blot results showing the expression level of ACSM5 in LFCs from HLF patients with the treatment of pCMV-DNMT1 or empty plasmid. Data were presented as the mean ± SME of three independent experiments. Two-tailed paired t-test, #*P* <0.05, ##*P* <0.01.

**Additional file 4: Supplementary Fig. 4** Identification of the phenotype of cultured ligamentum flavum (LF) cells using immunofluorescence staining. Immunofluorescence staining for collagen III and fibronectin in cultured cells, scale bar=100 μm, *n*=3 experiments.

## Data Availability

The datasets used and/or analyzed during the current study are available from the corresponding author on reasonable request.

## Abbreviations

LFH: Ligamentum flavum hypertrophy
LDD: Lumbar degeneration disease
LSCS: Lumbar spinal canal stenosis
SNP: Single-nucleotide polymorphism
ECM: Extracellular matrix
qMSP: Quantitative methylation-specific analysis
WES: Whole-exome sequencing
GO: Gene ontology
KEGG: Kyoto Encyclopedia of Genes and Genomes
BP: Biology process
CC: Cell component
MF: Molecular function
