# Supplementary figures and images for: Integrative analysis of genome-wide DNA methylation and single-nucleotide polymorphism identified ACSM5 as a suppressor of lumbar ligamentum flavum hypertrophy
Source: Arthritis Res Ther. 2021 Sep 30;23:251. doi: 10.1186/s13075-021-02625-5 (PMC8482693; doi:10.1186/s13075-021-02625-5)

Figure S1

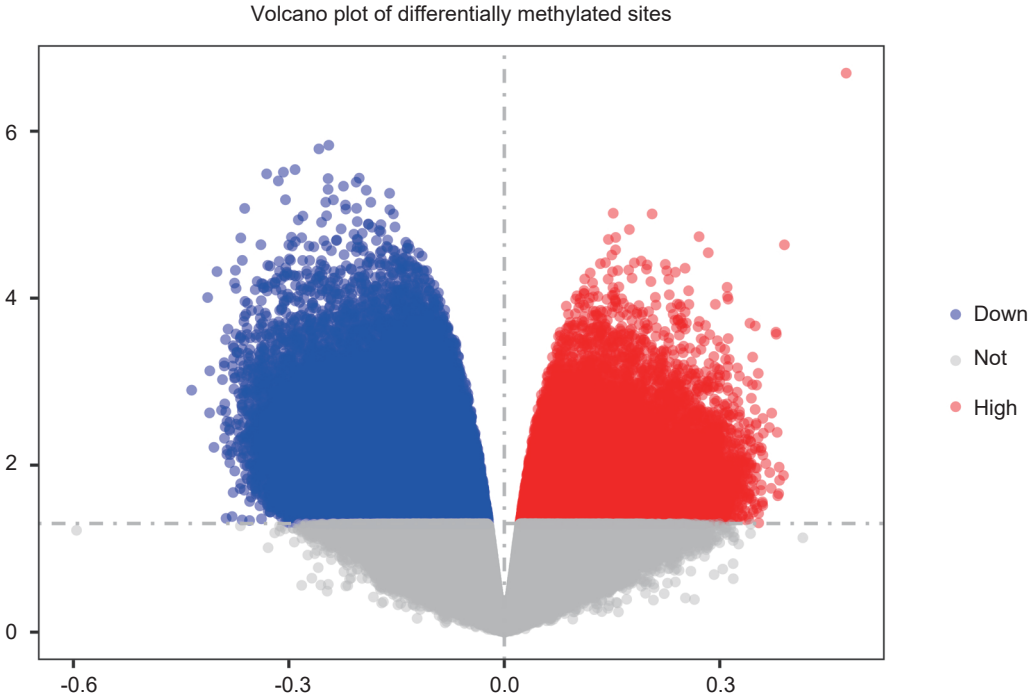

Supplement: Supplementary file 1 — Additional file 1: Supplementary Fig. 1 Volcano map showing the up-methylated sites and the down-methylated sites. The red dots indicate the methylation upregulation and the blue dots indicate the methylation downregulation in ligamentum flavum hypertrophy. [file 13075_2021_2625_MOESM1_ESM.pdf]

Figure S2

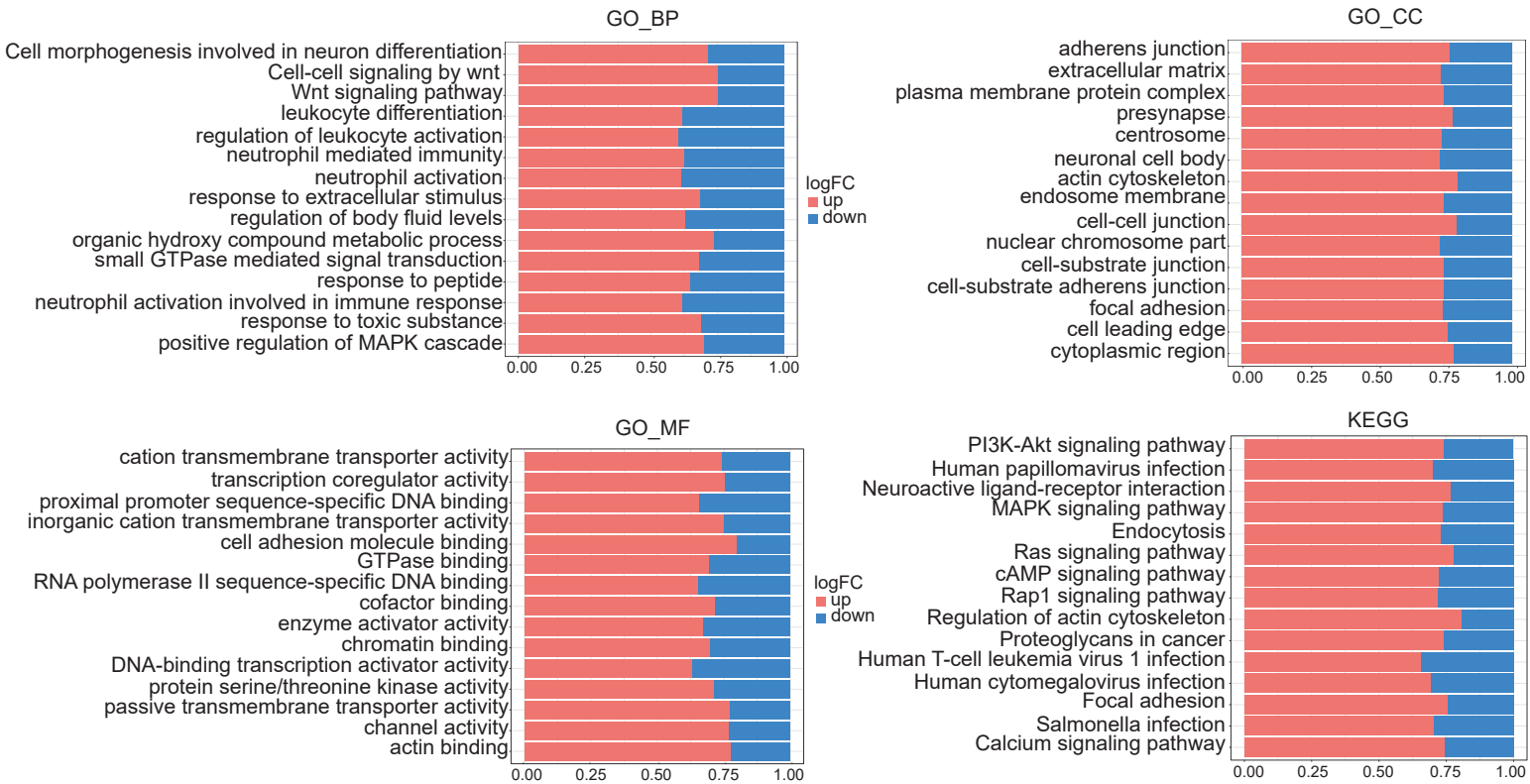

Supplement: Supplementary file 2 — Additional file 2: Supplementary Fig. 2 GO and KEGG enrichment analysis of the differentially methylated sites. Top 15 bio-functions or pathways enriched of the differentially methylated sites in GO_BP, GO_CC, GO_MF, and KEGG were shown, respectively. [file 13075_2021_2625_MOESM2_ESM.pdf]

Figure S3

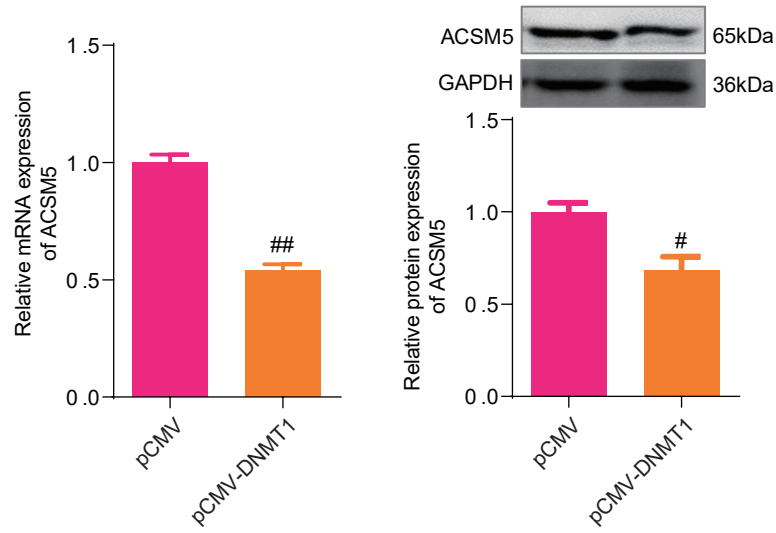

Supplement: Supplementary file 3 — Additional file 3: Supplementary Fig. 3 qPCR and Western Blot results showing the expression level of ACSM5 in LFCs from HLF patients with the treatment of pCMV-DNMT1 or empty plasmid. Data were presented as the mean ± SME of three independent experiments. Two-tailed paired t-test, #P <0.05, ##P <0.01. [file 13075_2021_2625_MOESM3_ESM.pdf]

**Figure S4**

**DAPI**

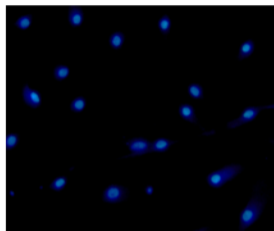

**Collagen III**

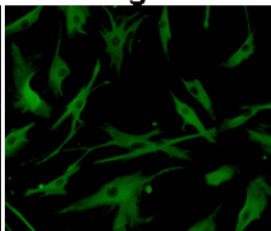

**Merge**

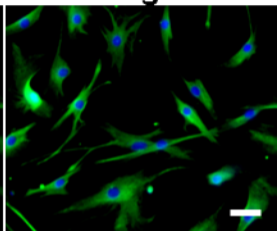

**DAPI**

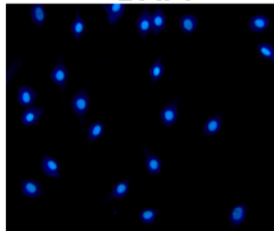

**Fibronectin**

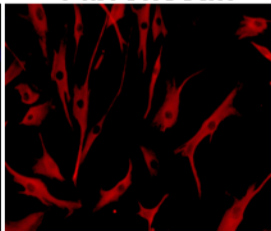

**Merge**

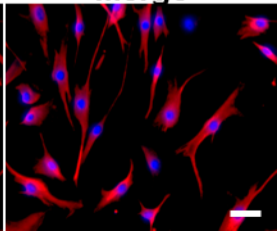

Supplement: Supplementary file 4 — Additional file 4: Supplementary Fig. 4 Identification of the phenotype of cultured ligamentum flavum (LF) cells using immunofluorescence staining. Immunofluorescence staining for collagen III and fibronectin in cultured cells, scale bar=100 μm, n=3 experiments. [file 13075_2021_2625_MOESM4_ESM.pdf]
